# Cocaine-mediated impact on HIV infection in humanized BLT mice

**DOI:** 10.1038/srep10010

**Published:** 2015-06-18

**Authors:** Sohn G. Kim, Emily L. Lowe, Dhaval Dixit, Cindy Seyeon Youn, Irene J. Kim, James B. Jung, Robert Rovner, Jerome A. Zack, Dimitrios N. Vatakis

**Affiliations:** 1 Department of Medicine, Division of Hematology-Oncology; 2 UCLA AIDS Institute, David Geffen School of Medicine at UCLA, Los Angeles, CA 90095; 3 Department of Microbiology, Immunology and Molecular Genetics; 4 Department of Molecular, Cell and Developmental Biology.

## Abstract

Cocaine abuse has been shown to have broad-ranging effects on human immunity. With regards to HIV infection, *in vitro* studies have shown that cocaine enhances infection of stimulated lymphocytes. Moreover, cohort studies in the pre- and post-HAART era have linked stimulant abuse with increased HIV pathogenesis. The latter data, however, have been undermined by a series of confounding factors underscoring the importance of controlled *in vivo* models to fully assess the impact of cocaine use and abuse on HIV infection and pathogenesis. Here, we have infected humanized mice with HIV-1 following acute cocaine exposure to assess the impact on infection. Stimulant exposure resulted in increased inflammatory cytokine expression, accelerated HIV infection, while blunting effector function of cytotoxic T lymphocytes. These data demonstrate cocaine’s multifactorial impact on HIV infection that extends beyond high-risk behavior.

Stimulants such as cocaine and methamphetamine are known co-factors in human disease pathogenesis and significant modulators of immune responses^1^,^2^,^3^,^4^,^5^,^6^. Cocaine has been shown to alter the phenotype and function of antigen presenting cells, macrophages, T cells, and the cytokines that they produce^1^,^2^,^3^,^7^,^8^,^9^,^10^,^11^,^12^,^13^,^14^,^15^,^16^,^17^,^18^,^19^,^20^,^21^,^22^,^23^,^24^,^25^,^26^,^27^. Many of these effects appear to directly impact on HIV infection and replication^8^,^26^,^28^. A compelling link between cocaine and HIV replication was first described *in vitro* studies in the early 1990’s^8^,^10^,^11^. Lymphocytes from healthy donors were stimulated in the presence or absence of cocaine, and evaluated for their ability to support HIV-1 infection and replication. HIV replication was enhanced up to 3-fold at cocaine concentrations between 0.3 pg/ml to 0.3 ng/ml (10^−9^ to 10^−6^ M). Subsequently, the same investigators found that addition of TGF-β to control cells induced similar changes in HIV replication, and the stimulatory effects of cocaine were completely blocked using neutralizing antibodies against TGF-β^8^,^10^. However, other cytokines and pathways have been implicated in the interaction between cocaine, T cells and HIV. For example, studies have demonstrated that cocaine can act via the intracellular sigma-1 receptor (σ1R) to up-regulate the expression of IL-10 and down-regulate the expression of IFN-γ, two cytokines centrally involved in HIV pathogenesis^24^,^26^,^29^. While the above studies point to a dampened inflammatory state, cohort studies reveal higher levels of inflammation demonstrated by increased levels of neopterin^3^, IFN-γ as well as TNF-α in the presence of cocaine^30^,^31^. Finally, our group recently demonstrated that cocaine exposure of quiescent T cells renders them permissive to HIV infection^32^. These effects were mediated in part by the σ1R and mainly through the dopamine D4 receptor (D4R)^32^. Therefore, cocaine exposure has both direct and indirect effects on HIV replication in CD4 T cells.

While *in vitro* studies have yielded valuable information on cocaine’s impact in HIV infection, they are limited in scope. Clinical and epidemiological studies have tackled the issue looking at various cohorts. While the data from a number of studies suggest that poor HAART adherence is the main factor enhancing HIV disease progression in cocaine users^33^,^34^,^35^,^36^,^37^, others have suggested that it may not a sole factor. In studies controlling for HAART adherence, cocaine users were more likely to have higher viral loads, display quicker and more robust viral rebounds upon cessation of HAART and exhibit higher morbidity^3^,^4^,^38^,^39^,^40^,^41^. While the data obtained from the cohort studies are quite valuable, they are confounded by factors including but not limited to HAART adherence, multidrug use and co-infections such as HCV and/or TB. Meanwhile, early chimeric mouse models were used to develop more controlled *in vivo* systems to study the effects of cocaine on HIV infection. In these studies, systemic daily administration of cocaine significantly increased the percentage of HIV-infected human lymphocytes and dramatically increased total viral loads^22^,^26^. However, the lack of peripheral reconstitution in these animals prevented more extensive studies on HIV pathogenesis and drug abuse. Advances in the development of humanized mice have allowed for such in-depth analysis of HIV pathogenesis and latency^42^,^43^ as well as immune function^44^,^45^. To this end, we have employed the BLT humanized mouse model to comprehensively assess the impact of cocaine on HIV infection. Following a 5-day cocaine pre-treatment, subsets of mice were infected with HIV-1_89.6_ followed by continuous cocaine administration. Mice were sacrificed two weeks after infection, at which we collected various tissues to measure levels of infection and examine other immunological parameters. Based on our data, pretreatment with cocaine resulted in an increased expression of inflammatory cytokines leading to accelerated HIV infection. Moreover, HIV infected, cocaine treated animals continuously expressed virus in peripheral blood and their effector activity of cytotoxic T cells was impaired. Our data demonstrate that cocaine exposure enhances early acute HIV infection both directly, as shown in our previous *in vitro* studies, and indirectly through changes in the immune phenotype of T cells.

## Results

### Acute exposure of humanized mice to cocaine results in increased immune activation

**Figure 1:**
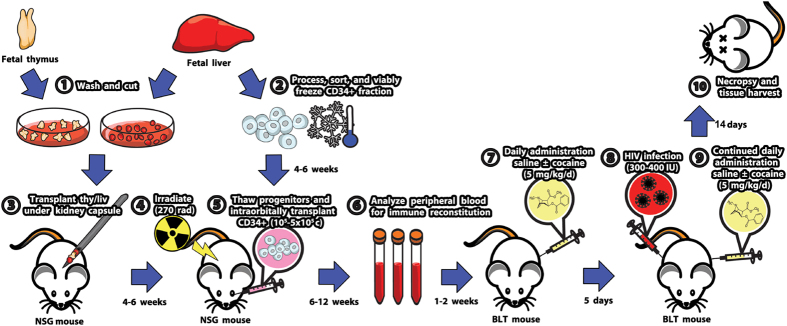
A schematic diagram of the experimental set up. BLT mice were generated and treated as described in the Materials and Methods section. Cocaine treated and untreated mice were sacrificed at two weeks after infection at which we harvested blood, spleen and thymus to assess levels of HIV infection and characterize immune cells.

The latest advances in humanized mice have led to the development of models that closely mimic human immunity and allow for the study of chronic infections. Using the BLT humanized mouse model, we examined *in vivo* the impact of cocaine exposure on HIV infection. Chimeric mice were constructed as previously described^44^,^45^ and shown in Fig. 1. Following reconstitution with human cells, cohorts were separated into 4 different groups based on treatment: (i) **NON** (saline, no HIV), (ii) **COCAINE** (cocaine **5 mg/kg/day**, no HIV), (iii) **HIV** (saline, HIV), and (iv) **COCAINE-HIV** (cocaine **5 mg/kg/day**, HIV-1_89.6_ 300–400 IU). We treated mice with 5 mg/kg of cocaine or saline every day for 5 days by intraperitoneal injection. At the end of 5 days, the **HIV** and **COCAINE-HIV** groups were infected intraperitoneally with HIV-1_89.6_. Cocaine or saline administration continued for 2 weeks after infection at which point mice were sacrificed to assess levels of infection and lymphocyte phenotype in various tissues (Fig. 1).

**Figure 2:**
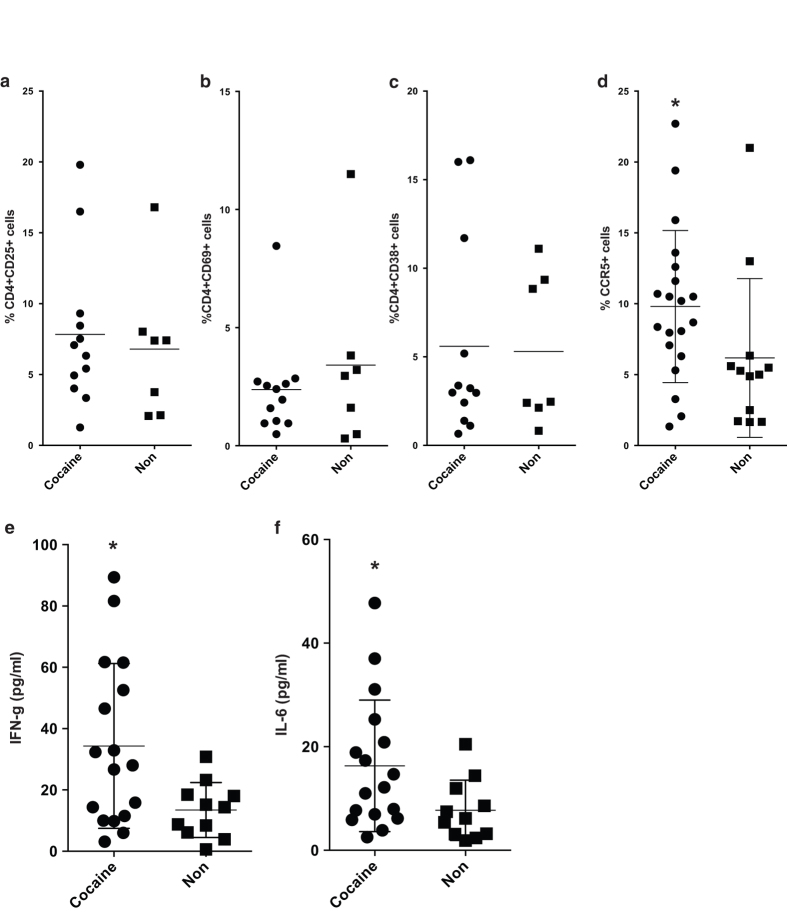
Five day acute cocaine exposure results in subtle but significant changes in CD4 T cell phenotype. (**a-c**) Blood samples collected after 5 days of cocaine exposure were used for flow cytometric analysis of T cell activation markers on CD4 T cells. There were no changes on the expression of CD25, CD69 and CD38. (**d**) Blood samples were also tested for CCR5 expression. The data are from three mouse cohorts. Cocaine treated animals demonstrated increased levels of the receptor (Cocaine – n = 20, Non – n = 12). (**e-f**) Plasma from the same mice was collected and used to examine levels of Th1/Th2/Th17 cytokines (Cocaine – n = 20, Non – n = 12). For all figures, *p < 0.05, **p < 0.01, using Mann-Whitney or Kruskal-Wallis test (for 3 or more groups).

To assess the effect of acute cocaine exposure prior to infection, blood samples were collected from mice and analyzed by flow cytometry. Based on our results, expression of CD25, CD69 and HLADR on CD4 T cells (Fig. 2a–c) was not altered in cocaine treated animals, a phenotype seen in our previous *in vitro* studies^32^. However, cocaine exposure resulted in upregulation of CCR5 expression on CD4 T cells, a phenotype previously seen in our *in vitro* studies (Fig. 2d)^32^. There was no effect on CXCR4 expression (data not shown). Furthermore, we collected blood samples to assess plasma cytokine levels. Cocaine treated animals demonstrated elevated levels of IFN-γ and IL-6 (Fig. 2e,f). These data suggest that exposure of humanized mice to cocaine promotes an inflammatory state capable of facilitating HIV infection.

### Cocaine treated animals demonstrated increased kinetics of HIV infection

**Figure 3:**
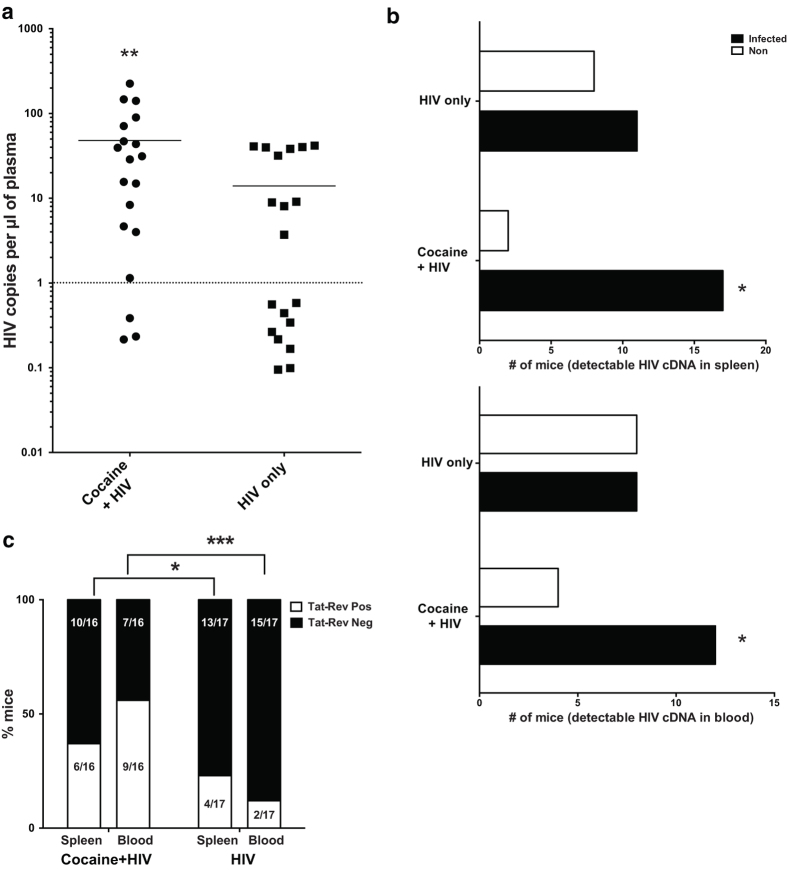
Acute cocaine exposure results in accelerated HIV infection of humanized mice. (**a**) After 5-day treatment, mice were infected with HIV_89.6_ followed by continued treatment with cocaine or saline for two more weeks. At the end of two weeks, mice were sacrificed; plasma samples were tested for levels of viral RNA. Cocaine treated mice had higher viral loads and a higher fraction of them had detectable levels of virus. (**b**) Samples from spleen and blood were tested for levels of viral cDNA the proportion of mice with detectable levels of viral cDNA were higher in the cocaine treated animals. (**c**) A higher fraction of cocaine treated animals had detectable levels of tat-rev mRNA in both spleen and blood. For all figures, *p < 0.05, **p < 0.01, using Mann-Whitney or Kruskal-Wallis test (for 3 or more groups). For proportion data, we used a Chi-square test.

At the end of the 5-day drug pre-treatment, **HIV** and **COCAINE-HIV** mice were infected with HIV-1_89.6_ (Fig. 1). Cocaine administration continued for the treated animals as indicated above. Two weeks after infection, the mice were sacrificed to assess the rate and level of infection. At this early timepoint using this route of inoculation, levels of infection in untreated humanized mice are typically low or undetectable^46^. Plasma samples were collected from all mice to measure viral loads. Based on our data, the **COCAINE + HIV** group had higher levels of viral loads than the **HIV** group (Fig. 3a). Most importantly, a higher fraction **(9/19)** of the untreated mice tested below the limit of detection for viral loads, while in the cocaine treated group only **3/19** had undetectable viral loads. Thus, cocaine exposure accelerated HIV replication in treated mice. In addition to plasma viral loads, we examined the presence and levels of viral cDNA in the blood and spleen of these animals. As shown in Fig. 3b, a higher fraction of cocaine treated animals had detectable levels of viral cDNA in these tissues. Thus, our model, in support of cohort studies, demonstrates that cocaine exposure, in the absence of other confounding factors such as co-infection or other drug use, accelerates viral replication.

### Cocaine treated animals express higher levels of spliced viral mRNA in peripheral blood

To further characterize viral replication in our mouse cohorts, in addition to viral cDNA, we examined the levels of multiply spliced RNA in blood and spleen samples. We focused our analysis on the animals that had detectable viremia based on the viral load assays. Expression of multiply spliced viral RNA is associated with ongoing viral replication^47^. As the majority of lymphocytes in blood are resting, we expected, based on previous studies, to see very minimal levels of tat-rev mRNA^47^. While this was true in the untreated mice, a significantly higher fraction of **COCAINE + HIV** mice had detectable tat-rev mRNA in the blood (Fig. 3c). The patterns were similar but not as striking in the spleen. This could be attributed to the presence of other activation signals present in a densely packed tissue such as the spleen that would allow for ongoing replication^48^. Thus, at this early stage of the infection, in the presence of cocaine there are higher levels of spliced viral RNA which are potentially contributing increased viral spread.

### Cocaine presence in HIV infected animals dulls CTL function

**Figure 4:**
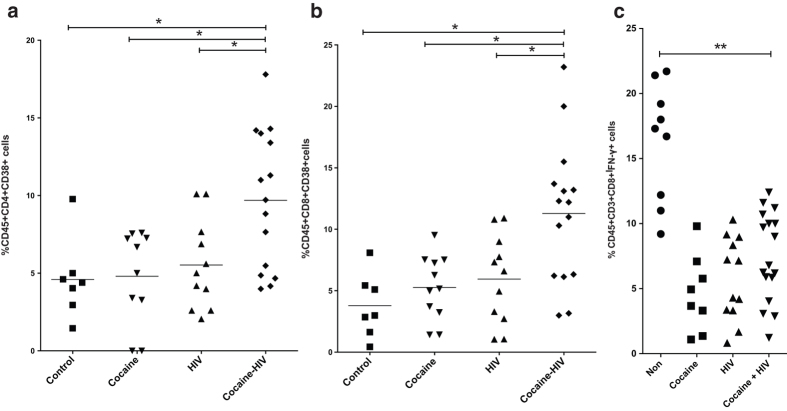
Increased activation persists in the cocaine treated, HIV infected animals. Splenocytes from the sacrificed mice were also used for flow cytometric analysis of lymphocyte phenotype. The **COCAINE-HIV** group of mice has higher levels of (**a**) CD4CD38 and (**b**) CD8CD38. (**c**) To assess functionality of CD8 T cells, we stimulated *ex vivo* splenocytes in the presence of GolgiPlug to measure levels of IFN-γ production. The COCAINE-HIV group had comparable levels to **HIV** despite the higher numbers of CD8CD38 T cells. For all figures, *p < 0.05, **p < 0.01, using Mann-Whitney or Kruskal-Wallis test (for 3 or more groups).

To further characterize the impact of cocaine on immunity during early acute HIV infection, we analyzed tissue samples for various immunological parameters such as T cell activation and plasma cytokine levels. Splenocytes of humanized mice with detectable viral loads were analyzed for T cell activation. Based on our data, the **COCAINE-HIV** group had elevated CD4+CD38+ T cells (Fig. 4a). Interestingly, the levels of activated CD8+CD38+ T cells were also higher in these animals (Fig. 4b). Since we detected increased levels of CD8+CD38+ T cells in the **COCAINE-HIV** group, we examined whether this increased activation would translate to robust effector cytokine release after *ex vivo* stimulation. This would suggest that the effector functions during early acute infection in the presence of cocaine may not be impaired. Thus, we stimulated *ex vivo* splenocytes from these cohorts using PMA/Ionomycin for 3 days. The **COCAINE-HIV** group did not exhibit significantly higher levels of IFN-γ production from the HIV only group despite the more activated phenotype (Fig. 4c).

**Figure 5:**
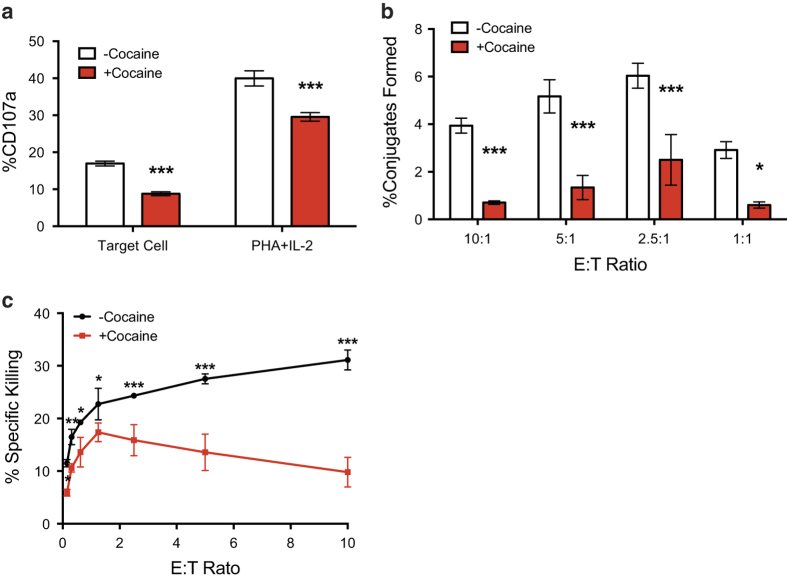
HIV-specific CTL are deficient in their ability to degranulate CD107a, form conjugates and kill HIV-infected cells upon treatment with cocaine. (**a**) HIV-specific CTL were co-incubated with HIV-expressing U1 cells at an 1:1 effector to target ratio or were treated with 10 μg/ml PHA and 100 U/ml IL-2 for 6 hr in the presence of protein transport inhibitors and antibodies to CD107a. Cocaine treatment of CTL resulted in a two-fold reduction in CD107a degranulation when CTL were co-cultured with HIV-infected cells and 26% less CD107a degranulation when CTL were maximally stimulated with PHA and IL-2 treatment. (**b** and **c**) HIV-specific CTL were co-incubated with HIV-expressing U1 cells at the defined effector to target ratios for 4 hr and subsequently assessed for their ability to induce (**b**) conjugate formation with or (**c**) apoptosis in target U1 cells. Cocaine treatment of CTL resulted in significantly impaired conjugate formation at all E:T ratios tested, which was further underscored by the significant loss in apoptosis induction of U1 target cells. Statistical significance was assessed by two-way ANOVA analysis, *p < 0.05, **p < 0.01, ***p < 0.001.

To further confirm our observations that CTL activity is dampened due to cocaine exposure, we carried out a series of *in vitro* experiments to determine if cocaine can directly impact CTL effector function such as killing HIV infected targets. To this end, we generated transgenic CTL to express a TCR specific for the HIV Gag epitope SL9^49^. These CTLs were then exposed to cocaine for 3 days as previously described^32^ and used in a series of assays to assess their effector activity. More specifically, these effectors were co-incubated with labeled, HIV infected U1 targets to measure their cytolytic activity. As shown in Fig. 5A, CTLs treated with cocaine demonstrated decreased levels of CD107a degranulation. Moreover, we observed decreased levels of conjugate formation (Fig. 5b), an indication that CTLs are targeting antigen expressing targets. Finally, as expected, the cocaine treated CTLs failed to effectively kill the HIV-infected U1 cells (Fig. 5c). Thus, despite an activated phenotype the effector function of CTLs seems to be severely blunted.

## Discussion

Cocaine abuse has been linked to increased HIV pathogenesis, disease progression and morbidity. While there is a plethora of epidemiological studies supporting this phenomenon, this valuable information is hampered by a series of cofounding factors such as concurrent drug use and co-infections^4^. Moreover, these studies do not go beyond a mere measurement of indicators such as viral loads and CD4 counts. Thus, the mechanisms behind the potentiating effects of cocaine in HIV infection have not been fully examined, thus, hampering potentially more effective interventions for drug using populations.

One way of addressing the challenges as well as the limited breadth of data is the development of controlled *in vivo* models that would allow us to study the relationship between HIV infection and cocaine abuse. Previous studies have used chimeric mouse models demonstrating the potentiating effect of cocaine^22^,^26^. However, these models were limited as they lacked peripheral immune reconstitution. To this end, we decided to use the BLT humanized mouse model to examine the effects of cocaine on HIV infection. In this model, there is full reconstitution of all immune lineages, which allows for the study of HIV infection and immune responses. The model has been used in a variety of settings such as cancer, gene therapy and vaccine development^42^,^43^,^44^,^45^,^46^,^49^,^50^,^51^,^52^.

Following the generation of humanized mice, we exposed a subset of them to cocaine for 5 days. On day 5, the animals were bled to assess immune activation and a subset of them was infected with HIV. Untreated, HIV infected animals were also included alongside untreated and cocaine only treated groups. Five-day acute pre-exposure of humanized mice to cocaine resulted in an overall increase of immune activation. However, as seen in our recent *in vitro* studies, the level and type of activation does not conform to the typical responses seen after TcR engagement. More specifically, we did not observe upregulation of classical T cell activation markers; we observed elevated expression of IFN-γ and IL-6. These data were in agreement with previous studies that have shown in cocaine users elevated serum neopterin as well as IFN-γ and IL-6 expression^3^,^30^. In addition, we observed an upregulation of CCR5 expression in CD4 T cells, a pattern also seen *in vitro*. Thus, based on our previous work^32^ and the current studies, the presence of cocaine directly and indirectly allows for the virus to more efficiently infect its target cell populations and replicate.

Based on the above, we anticipated HIV infection kinetics would be enhanced. As shown in our data, the presence of cocaine accelerated the progression of HIV infection as a higher fraction of animals had detectable levels of virus in serum. This was further supported by the detectable levels of viral cDNA in both spleen and blood samples. Thus, this supports the notion that cocaine alone can accelerate HIV infection^4^.

Furthermore, the presence of cocaine result in increased levels of detectable spliced viral mRNA. The latter correlates well with efficient viral spread^47^. Based on our data, we saw increased levels of tat-rev mRNA in both blood and spleen in the cocaine treated animals as opposed to the non-treated. This is quite interesting as CD4 T cells in blood are normally in a resting state and studies have shown that the majority of them are not expressing detectable levels of multiply spliced viral mRNA^47^. Thus, these data suggest that in the presence of cocaine, there are higher levels of viral expression allowing for more efficient viral replication.

To elucidate other means by which cocaine could influence HIV infection, we analyzed the immunological profiles of immune cells and plasma cytokine levels in the HIV infected cohorts. The cocaine exposed animals had higher levels of CD4+CD38+ T cells pointing to a more activated state that would support efficient and continuous HIV replication. Interestingly, these mice had elevated numbers of CD8+CD38+ T cells. Therefore, these cells should be able to limit spread of infection by killing infected targets in early acute infection. However, when we activated splenocytes *ex vivo*, we saw that the CD8 T cells did not express higher levels of IFN-γ in the **COCAINE-HIV** group suggesting that effector responses may be blunted by the presence of cocaine. Our *in vitro* assays demonstrated that cocaine can directly diminsh CTL effector function. The mechanisms of this phenotype will be further investigated.

In conclusion, using the BLT humanized mouse model, we demonstrate that acute cocaine exposure results in accelerated HIV replication, increased viral expression, enhanced inflammation, and yet potentially blunted CTL activity. More importantly, this study is a proof of principle that we can use the BLT mouse model to explore in full all aspects of cocaine abuse and its impact on HIV pathogenesis and disease progression. Finally, these studies demonstrate that cocaine exposure does not simply enhance HIV infection through high risk behavior, but also importantly through significant changes on the physiology and function of human immune cells that allow the virus to replicate and spread efficiently.

## Materials and Methods

### Ethics statement

Peripheral blood mononulear cells was obtained at the University of California, Los Angeles in accordance with UCLA Institutional Review Board (IRB) approved protocols under written informed consent using an IRB-approved written consent form by the UCLA/CFAR Virology Laboratory and was distributed for this study without personal identifying information. Human fetal tissue was purchased from the UCLA/CFAR Gene Therapy Core, was obtained without identifying information and did not require IRB approval for use. Animal research carried out in this manuscript was performed under the written approval of the UCLA Animal Research Committee (ARC) in accordance to all federal, state, and local guidelines. Specifically, the experiments were performed strictly according to the guidelines in The Guide for the Care and Use of Laboratory Animals of the National Institutes of Health and the accreditation and guidelines of the Association for the Assessment and Accreditation of Laboratory Animal Care (AALAC) International under UCLA ARC Protocol Number 1997-176-53.

### Cells

U1 cells (NIH AIDS Reagent Program) are a subclone of the promonocytic cell line U937 that is chronically infected with HIV and makes replication competent virus upon activation^53^. J.RT3-T3.5 (ATCC, Manassas, VA) are a subclone of leukemia T cell line Jurkat mutated in the T cell receptor beta chain locus such that these cells fail to express surface CD3 and TCR. U1 and J.RT3-T3.5 cells were maintained in culture media containing RPMI 1640 (Invitrogen, Life Technologies, Carlsbad, CA) supplemented with 10% fetal bovine serum (Omega Scientific, Tarzana, CA) and 100 U/ml penicillin and 100 μg streptomycin (GIBCO, Life Technologies).

### Virus stocks

For all the studies, we used HIV-1_89.6_, a dual tropic HIV strain that infects cells expressing CXCR4 and/or CCR5 co-receptors. The strain was selected to ensure infection of all susceptible cell populations. Stocks of HIV-1 molecular clone 89.6 were obtained from 24-h harvests of supernatants from infected CEMx174. Supernatants were filtered and treated with DNase (2 μg/ml) (Worthington, Lakewood, N.J.) for 30 min at room temperature in the presence of 0.01 M MgCl_2_. Viral infectivity was determined by limiting dilution titration on GHOST (3) X4/R5 (NIH AIDS Reagent Program). Lentiviral vectors expressing a TCR specific for the HIV Gag epitope SL9 were produced by calcium phosphate transfection of 293FT cells with the pCCL.PPT.SFFV.1.9.IRES. dLNGFR^54^ in conjunction with the lentiviral packaging vectors pMDLg/pRRE and pCMV-VSV-G. Supernatants were harvest on day 2 and passed through a 0.45 micron filter and concentrated by ultracentrifugation. Infectivity was assessed by titration of lentiviral vectors on J.RT3-T3.5 cells and flow cytometric analysis for the co-expression of SL9TCR (SL9 iTAg MHC Class-I tetramer-PE, Beckman Coulter, Brea, CA) and dLNGFR (CD271-FITC, Stem Cell Technologies, Vancouver, British Columbia, Canada).

### Flow cytometry

Fluorochrome-conjugated mAb, specific for human CD4, CD8, CD25, CD38, CD45, CD69, CCR5, CXCR4, HLA-DR were obtained from BD Biosciences (San Diego, CA). For each analysis, cells were labeled with mAb, fixed with 2% paraformaldehyde, and analyzed using BD LSRII Fortessa flow cytometer (BD Biosciences) using FACSDiva software. Gating on human anti-CD45-stained cells was used to exclude contaminating murine cells. Subsequent analyses were performed using FlowJo software (Tree Star, Ashland, OR).

### Serum cytokine assay

Blood samples from our cohorts were recovered via puncture of the retro-orbital venous plexus using EDTA coated capillaries. Samples were spun at 3000 rpm for 5 min and serum was collected. Cytokine levels were determined using a cytometric bead array assay (BD CBA Human Th1/Th2/Th17 Cytokine Kit, BD Biosciences) specific for human IL-2, IL-4, IL-6, IL-10, TNF, IFN-γ, and IL-17A. Samples were acquired on a BD LSR Fortessa flow cytometer (BD Biosciences) with FACSDiva software, and subsequent analyses were performed using FCAP Array software.

### Generation of BLT mice and treatment

NOD.Cg-Prkdc^scid^ Il2rg^tm1Wjl^/SzJ (NSG) mice were initially purchased from Jackson Laboratories and bred and maintained under laminar flow conditions in the Mouse/Human Chimera Core Facility at the University of California, Los Angeles (UCLA). Humanized mice were prepared as previously described^44^. Mice were then monitored for human cell engraftment 6 - 10 weeks post-injection. Upon reconstitution the animals were treated with cocaine for 5 days. Cocaine hydrochloride (5 mg/ml in saline) was obtained from the National Institute on Drug Abuse [NIDA; National Institutes of Health (NIH), Bethesda, MD] and diluted in saline prior to use. This dose of cocaine was selected on the basis of prior dose/response experiments (0.1, 5 or 10 mg/kg)^7^,^55^ in which the 5 mg/kg dose was shown to have no effects on engraftment yet significantly enhanced HIV infection^22^. Cocaine was delivered by intraperitoneal (i.p.) injection beginning 5 days pre-infection (4 - 8 weeks post human tissue implantation). After the 5-day pretreatment, a subset of animals was infected with HIV-1_89.6_, followed by continuous cocaine administration until day 14 post-infection when the animals were sacrificed. Each experiment utilized humanized mice that were made from human tissue from the same donor and the donor tissue was unique experiment to experiment. Animals exhibiting any symptoms of graft versus host disease (GVHD) are removed from our analyses.

### Viral load assay

Peripheral blood was collected by cardiac puncture and transferred into microcentrifuge tubes containing 330 mM EDTA. Viral RNA was extracted from plasma using the QIAamp Viral RNA Mini Kit (Qiagen). Quantitative RT-PCR was performed using the following primers/probe specific for gag sequences: NG1F (position 453–480) 28 bp (5′-GAGCTAGAACGATTCGCAGTTAATCCTG-3′), NG1R (position 570–534) 37 bp (5′-ATAATGATCTAAGTTCTTCTGATCCTGTCTGAAGGGA-3), NG1Z probe (position 482–520) 39 bp (FAM-5′ -CCTTTTAGAGACATCAGAAGGCTGTAGACAAATACTGGG-3-BHQ). Reverse transcription was performed using the Superscript II kit (Invitrogen). Real-time, quantitative PCR was performed on a BioRad CFX96 thermocycler. Results from samples were interpolated within the quantitation derived from the RNA standards.

### Tat/rev real time RT-PCR

Expression of viral genomic RNA was measured by quantitative real-time RT-PCR and compared to *in vitro*-transcribed RNA standards specific for multiply spliced Tat-Rev RNA as previously described^56^. The 18S primer/probe set was obtained from Applied Biosystems specific for eukaryotic 18S rRNA as an endogenous control to allow relative gene expression quantification. The reaction conditions were carried out using the iScript One-Step RT-PCR Kit for Probes (Bio-Rad)^56^.

### Quantitative real time PCR

We used quantitative real-time PCR to detect the presence of viral DNA as previously described^56^. Briefly, cells were harvested and DNA was subsequently isolated to be used in a quantitative real time PCR using primers specific for HIV-1 sequences^56^. A primer-probe pair specific for the human β-globin gene was utilized to determine the input of cellular DNA as an endogenous control to allow relative gene quantification^56^. The reactions were carried out using the TaqMan Core Reagents Kit (ABI Biosystems)^56^.

### Intracellular cytokine assay

1 × 10^6^ cells/well were seeded in 48-well plates in RPMI-10 media and stimulated for 6 hours with PMA/Inonomycin at 37 °C, 5% CO2. Following incubation, the cells were washed and stained with antibodies against CD3, CD4, CD8, CD45, CD56 and IFN-γ (BDBiosciences) to measure levels of cytokine release. Samples were run on an LSRII Fortessa (BD Biosciences) and analyzed using FlowJo.

### Generating HIV-specific CTL

CD8+ T cells were isolated by magnetic bead isolation (EasySep Human CD8+ Selection Kit, Stem Cell Technologies) from the PBMC of healthy donors obtained by the UCLA CFAR Virology Core and activated overnight in 50 ng/ml anti-CD3 (clone OKT3, Imgenex, San Diego, CA) and 300 U/ml IL-2 (NIH AIDS Reagent Program). Activated CD8+ were then transduced with a lentiviral vector containing HIV SL9-specific TCR and cultured in AIM-V media (Invitrogen, Life Technologies) supplemented with 5% human AB serum (Omega Scientific), 20 ng/ml IL-7 and 20 ng/ml IL-15 (both Invitrogen, Life Technologies). CD8+ T cells expressing the SL9-specific TCR were purified by magnetic bead separation based on dLNGFR expression (EasySep Human CD271 Selection Kit, Stem Cell Technologies) and confirmed >85% by flow cytometric analysis.

### Cytotoxicity Assay

Purified SL9TCR-expressing CD8+ T cells were incubated in culture media (described above) for 3 days with 10 μM cocaine (NIDA Drug Supply Program) or not. On the second day, they were labeled with 2 μM Vybrant CFDA-SE (Invitrogen, Life Technologies) according to manufacturer’s suggested protocol and maintained in fresh culture media with 10 μM cocaine overnight. On the same day, target U1 cells were labeled with 5 μM CellTrace| Violet (Invitrogen, Life Technologies) according to manufacturer’s suggested protocol and activated to produce HIV with 10 μM prostratin (LC Labs, Woburn MA). The following day, cocaine or mock treated CTL were co-incubated with prostratin-activated U1 cells for 4 hr at the described effector to target ratios. Target cell sensitivity to CTL was assessed by the intracellular expression of cleaved caspase 3 via flow cytometric analysis. Percent specific killing was assessed by subtracting the %cleaved caspase 3 in target cells cultured with CTL from the %cleaved caspase 3 in target cells cultured alone. Conjugates of effector CTL and target U1 cells were assessed by flow cytometric analysis of Vybrant CFDA-SE and CellTrace Violet double positive for cells.

### IFN-γ Production and CD107a Degranulation Assays

Purified SL9TCR-expressing CD8+T cells were incubated in culture media (described above) for 3 days with 10 μM cocaine or not. On the second day, they were labeled with 2 μM Vybrant CFDA-SE (Invitrogen, Life Technologies) according to manufacturer’s suggested protocol and maintained in fresh culture media with 10 μM cocaine overnight. On the same day, target U1 cells were labeled with 5 μM CellTrace Violet (Invitrogen, Life Technologies) according to manufacturer’s suggested protocol and activated to produce HIV with 10 μM prostratin (LC Labs, Woburn MA). The following day, cocaine or mock treated CTL were co-incubated with prostratin-activated U1 cells for 6 hr at an 1:1 effector to target ratio in the presence of GolgiStop and GolgiPlug (BD Biosciences, San Jose, CA) and PerCP/Cy5.5-anti-CD107a (1:50, Biolegend, San Diego, CA). Some CTL were also treated with phytohemagglutinin, PHA, (10 μg/ml) and IL-2 (100 U/ml) to induce maximal expression of CD107a and IFN-γ. After 6 hr, IFN-γ expression was assessed by intracellular cytokine staining.

### Statistical analysis

All statistical analyses were carried out using GraphPad Prism 6. For two groups, we used Mann-Whitney test or one-tailed Student’s t-test, while for groups of 3 or more we used a Kruskal-Wallis (with Dunn’s multiple comparisons test) or one-way ANOVA (with a Tukey post-test). For most of our analysis, unless otherwise indicated, we ran non-parametric tests. For our proportion analysis, we used a Fisher’s exact test.

## Additional Information

**How to cite this article**: Kim, S. G. *et al.* Cocaine-mediated impact on HIV infection in humanized BLT mice. *Sci. Rep.* 5, 10010; doi: 10.1038/srep10010 (2015).
